# Relationships between neurodivergence status and adverse childhood experiences, and impacts on health, wellbeing, and criminal justice outcomes: findings from a regional household survey study in England

**DOI:** 10.1186/s12916-024-03821-1

**Published:** 2024-12-18

**Authors:** Charley Wilson, Nadia Butler, Zara Quigg, David Moore, Mark Bellis

**Affiliations:** Liverpool John Moores University, Liverpool, UK

**Keywords:** Adverse childhood experiences, Neurodivergence, Health and wellbeing, Criminal justice

## Abstract

**Background:**

Evidence indicates that neurodivergent (ND) populations may be more at risk of experiencing adverse childhood experiences (ACEs), compared to neurotypical (NT) populations. However, this evidence has typically not examined a comprehensive set of ACEs and has only included ND individuals on the basis that they have a diagnosis. Very little research has examined the impacts of ACEs on negative adulthood outcomes for ND populations. The current study aimed to examine the associations between neurodivergence and experiences of ACEs, and the impact of being ND and experiencing ACEs on health, wellbeing, and criminal justice outcomes.

**Methods:**

From November 2023 to April 2024, a household survey using representative sampling was undertaken with 5395 residents of an English region aged 18 + years. Neurodivergence status was measured using one self-report item. Nine ACEs were measured using validated self-report items. Outcome measures included: poor general health, low mental wellbeing, ever being arrested, and ever being incarcerated. Multinomial regression models were used to examine relationships between neurodivergence status and ACEs. Binary logistic regression models were used to examine independent relationships between neurodivergence status and ACE count and each outcome measure. Generalised linear models with an estimated marginal means function were used to estimate the increased risk of each outcome for different combinations of neurodivergence and ACE count status (NT less than four ACEs (reference group), NT 4 + ACEs, ND less than four ACEs, ND 4 + ACEs).

**Results:**

A higher proportion of ND individuals experienced each ACE type than NT individuals. While controlling for sociodemographics, ND individuals were more likely to experience a greater number of ACEs than their NT peers. While controlling for sociodemographics, each outcome measure was more likely amongst those who were ND, and each outcome measure except for poor general health was more likely amongst those with higher ACE counts.

**Conclusions:**

The combination of being ND and experiencing ACEs could additively increase risks of experiencing poor wellbeing and criminal justice outcomes by a greater extent than expected. Preventing and responding to ACEs in ND populations should be a priority to reduce risks of poor health, wellbeing, and criminal justice outcomes in this population.

**Supplementary Information:**

The online version contains supplementary material available at 10.1186/s12916-024-03821-1.

## Background

Neurodiversity describes the variations that exist between different types of brains, with neurodivergent (ND) individuals having brains that work in different ways to the neurotypical (NT) majority [1]. ND populations are made up of individuals who may have a range of different diagnoses (e.g. autism, attention deficit hyperactivity disorder (ADHD), dyscalculia, dyslexia, dyspraxia, obsessive compulsive disorder (OCD), and Tourette’s syndrome). Some ND individuals will have a diagnosis of one of these, some will be self-diagnosed, and some will not have any kind of recognition, yet will still experience aspects of life differently to their NT peers.

Research has highlighted that across the lifecourse ND populations may have different physical and mental health outcomes to their NT peers. For example, amongst some ND populations there are heightened levels of a range of physical health conditions, engagement with different health risk behaviours, such as alcohol use and drug use, experiences of reduced wellbeing, co-occurring psychiatric conditions such as depression and anxiety, and experiences of suicidality [2–8]. Critically, such physical and mental health challenges may create difficulties for ND people in how they engage with society, including in education, in employment, and in socialising, and may have implications for offending behaviours [4, 5]. For example, research indicates that ND individuals are more likely to be excluded from school than their NT peers and may be over-represented within youth justice and criminal justice systems [6, 9–11]. Across the lifecourse all of these factors may exacerbate any existing health and wellbeing concerns for ND people.

Evidence also indicates that some ND populations may face heightened levels of adverse childhood experiences (ACEs) and associated trauma [12–15]. This may be due to for example abuse occurring under the guise of ‘correction’ of behaviours which deviate from what is perceived as the norm, or reduced access to help due to communication differences to break away from abusive environments. ACEs include violence such as physical, verbal, and sexual abuse and neglect, and other household-level stressors such as parental separation, alcohol or drug harms, mental health difficulties, incarceration, and witnessing violence in the household [16]. Evidence indicates that there are significantly heightened levels of negative health, wellbeing, and social outcomes in adulthood for those who experience ACEs. A meta-analysis of studies examining relationships between experiencing ACEs and adulthood health outcomes showed that those who suffered four or more ACEs were more likely to experience mental health difficulties, engage in health risk behaviours, and be involved in violence as a victim or perpetrator, compared to those who experienced no ACEs [17]. Further, studies from across England and Wales have shown that those with four or more ACEs are more likely to be incarcerated than those with no ACEs [18, 19]. Research from England and Wales has also shown that there are increased risks of negative outcomes even for those who experienced only one type of ACE [20].

Overall, few studies have examined the prevalence of ACEs in ND populations, compared to NT peers, using large representative general population samples of adults. The studies which have been conducted also often rely on parental reporting of ACEs for their child, and do not always measure a comprehensive number of ACEs, often excluding experiences of abuse and neglect [13, 15]. Studies are often conducted when individuals are still children, thus not covering the whole period of childhood and risk of exposure to ACEs, and in addition these studies may exclude the experiences of children who have not yet been identified as ND; this might be a particular challenge as the number of individuals being diagnosed in later life is growing as well as growing waiting lists for diagnosis [21]. Therefore, these factors may underestimate the true prevalence of ACEs in ND populations. Further, limiting research to only including ND children omits the longer-term impacts of ACEs on ND adults; this is part of a wider research challenge in that the experiences of ND adults are often overlooked.

ACEs can have a range of impacts on biology, including changes to brain development, and alterations to the function of neurological, endocrine, and immune systems [16, 22]. Maladaptation of the stress response system in response to ACEs is likely to be one key process underlying the relationships between negative adulthood outcomes and experiences of ACEs. Repeated exposure to stressors in childhood can change the way that levels of hormones, such as adrenaline and cortisol, are regulated, resulting in hyper- or hypo-responsivity to release of these hormones, meaning that individuals may react disproportionately to potentially lower-level environmental stressors [16, 23]. Repeated or long-term activation of the stress response system also leads to an increased allostatic load, promoting the earlier development of non-communicable diseases, such as cardiovascular disease and cancers [16, 22].

Many of the challenges faced by ND individuals will be similar to their NT peers. However, due to differences between NT and ND populations in elements such as sensory processing, communication, social navigation, and emotional regulation, some ND individuals may also face a number of situations which act as additional stressors that NT individuals will not necessarily experience as a significant stressor [4]. As such, there may be a greater allostatic load in individuals who are both ND and experience ACEs, creating a ‘double jeopardy’ which increases the risks of negative outcomes in this group to a greater extent than either experiencing ACEs or being ND alone [23]. Here, we could identify only one study that has previously examined outcomes in populations on the basis of neurodivergence and ACE count. This longitudinal study of twins conducted in Sweden found that ND adolescents who had experienced ACEs were significantly more likely to experience symptoms of mania than ND adolescents without ACEs, NT adolescents with or without ACEs [24]. More research is required to better understand the combination of neurodivergence and experiences of ACEs on population health and wellbeing and offending behaviours, particularly using broader outcome measures, rather than focussing on very specific outcomes that may impact limited proportions of the population (e.g. mania), and including individuals who are neurodivergent but do not have a formal diagnosis.

### Aims

The current study aims to examine the association between neurodivergence and experiences of ACEs, and the impact of being ND and experiencing ACEs on mental wellbeing, general health, and criminal justice outcomes.

## Methods

A cross-sectional survey of adults aged 18 + years who are residents of households across an English region with five local authorities was carried out from November 2023 to April 2024. The study was conducted collaboratively by Liverpool John Moores University and the regional Violence Reduction Partnership, a public sector body with a focus on the prevention of violence. The study aimed to understand the nature and extent of violence and inform prevention activity [25]. A private market research company was commissioned to carry out the data collection.

### Sampling

The study utilised quota sampling to select 110 Lower Super Output Areas (LSOAs; small geography areas of similar population size with around 1500 residents) stratified by English Index of Multiple Deprivation (IMD; 26) quintile, age, and sex, from five local authorities. The IMD provides official measures of relative deprivation for LSOAs in England. Deprivation was measured using the English IMD [26] which comprises of a combination of 39 indicators across seven different domains of levels of relative deprivation including income; employment; health deprivation and disability; education, skills, and training; crime; barriers to housing and services; and living environment. An overall measure of deprivation is calculated for each LSOA in England; LSOAs can then be categorised into deprivation quintiles for an area based on their ranking in the IMD. The achieved sample size was 5395. This sample size was selected as 500 individuals with four or more ACEs were needed to meet the wider aims of the project, and other studies [18] suggested that this sample size would be adequate for this.

### Recruitment

Postal letters were sent to randomly selected households within each selected LSOA. Contacted households were given information about the study, including that participation was entirely voluntary, and that all data collected would be confidential and anonymous. Contacted households were given the option to take part in the survey online. If a member of the household did not complete the survey online, and had not opted out of the study, then a trained interviewer from the research company would visit their household so that they could take part in the survey in-person. Household visits were made on all days of the week and at varying times of day from 9 am to 9 pm. If interviewers did not receive an answer on the first time of visiting a household, interviewers would call back up to five times on different days and different times. If an individual was ineligible or refused to participate in the study, the interviewer recorded the outcome of the contact then moved on to the next randomly selected household.

Only one individual from each household was eligible to participate in the study, and individuals within a household who met the age and sex quotas were prioritised. If more than one individual in a household was eligible, the interviewers would ask for the person whose birthday is next to take part. The study utilised computer assisted personal interviewing technology, and for more sensitive parts of the survey computer assisted self-interviewing.

### Response rate

A total of 54,761 postal letters were sent to households in randomly selected LSOAs. From these letters, 467 households opted out of participating in the research. For the face-to-face element of the research, there were 6040 households that were visited where an eligible participant answered the door, of these 4180 completed the survey, giving a response rate of 69.2%. This response rate is within the range of participation rates (49.1–70.4%) for similar household surveys measuring the prevalence of ACEs in different areas across England and Wales [20]. Overall, 1215 participants (22.5%) completed the survey online from the postal letters and 4180 participants (77.5%) completed the survey face-to-face with trained interviewers.

### Measures

#### Neurodivergence

Participants were given a list of examples of neurodivergent diagnoses including autism, ADHD, OCD, dyslexia, dyspraxia, dyscalculia, and learning disabilities, and were asked ‘Which of the following best describes you?’: response options included: I have been diagnosed with a neurodivergent condition; I have self-diagnosed or suspect that I may have a neurodivergent condition; I have not been diagnosed and do not suspect that I may have a neurodivergent condition; prefer not to say. For analyses in the current paper, those who were either diagnosed neurodivergent, or self-diagnosed or suspecting, were combined into one group of neurodivergent individuals [27]. Those who answered prefer not to say were classed as missing data, and as such were not included in the analyses.

#### ACEs

ACEs were measured using validated items adapted (for relevance and cultural sensitivity) from previous surveys (18; Behavioral Risk Factor Surveillance System (BRFSS) questionnaire, [28]) and the Adverse Childhood Experiences International Questionnaire [29]. Items included whether the individual before the age of 18 years experienced physical, verbal, or sexual abuse; and household stressors including if their parents had separated, if they had witnessed domestic violence, and if they lived with anyone who had problems with alcohol or drugs, or was mentally ill, or was incarcerated. Respondents could answer yes, no, or prefer not to say to these items. As has been done in other studies [18, 29], the total number of these experiences was summed to give a total ACE count, which can be categorised into experiencing 0 ACEs, 1 ACE, 2–3 ACEs, and 4 + ACEs. To give a minimum count for each ACE, those who ‘preferred not to say’ (range: 4.8–7.7%) to an ACE item were recoded as no. ACE items from the 2010 BRFSS (these items are the same as those used in later versions, [28]) were shown to have acceptable internal reliability (Cronbach’s alpha = 0.78) in general population samples from the USA [30].

#### General health

The EQ-VAS (part of the EQ-5D-5L instrument; [31]) is a self-reported measure of general health on a vertical visual scale from 0 to 100, where 0 = ‘the worst health you can imagine’, and 100 = ‘the best health you can imagine’. Scores were dichotomised to indicate poor general health as more than one standard deviation (22.385) below the mean (73.210), thus poor general health was categorised as scores < 50.825. In the current study, the EQ-VAS was adapted, with the visual element of the scale removed, asking participants to instead indicate their score. Those who answered prefer not to say to this item were classed as missing data, and as such were not included in the analyses. The EQ-VAS shows sufficient construct validity in general population samples [32].

#### Mental wellbeing

Mental wellbeing was measured using the Short Warwick-Edinburgh Mental Wellbeing Scale (SWEMWBS; [33]). This is a validated scale including seven items about an individual’s current mental wellbeing, scored on a 5-point scale (1 = none of the time; 2 = rarely; 3 = some of the time; 4 = often; 5 = all of the time). Total scores on the SWEMWBS range from 7 to 35, with higher scores indicating higher levels of mental wellbeing. Raw scores are then converted to metric scores using a standard conversion table [33]. Scores were dichotomised to indicate low mental wellbeing as more than one standard deviation (5.1781) below the mean (24.9738), thus low mental wellbeing was categorised as scores of < 19.7957. Those who answered prefer not to say to any of the seven SWEMWBS items were classed as missing data, and as such were not included in the analyses. SWEMWBS shows high internal reliability (Cronbach’s alpha = 0.84) and high relative validity to the full WEMWBS scale (kappa = 0.79–0.85) in an English general population sample [34].

#### Arrest and incarceration

Participants were asked ‘Have you ever been arrested in the UK?’ and ‘Have you ever spent a night in prison or jail in the UK?’; response options were yes, no, and prefer not to say. Those who answered prefer not to say to arrest and incarceration items were classified as missing data and as such were not included in the analyses.

#### Sociodemographics

Sociodemographic characteristics controlled for in regression models included: sex (male, female), age (years: 18–24, 25–54, 55 +), ethnicity (White or other ethnicities), and deprivation quintile (1 most deprived; 5 least deprived).

### Data analyses

Analyses were undertaken in SPSS (v.28). Bivariate analyses using chi-squared tests were used to explore associations between neurodivergence status, different types of ACEs, and ACE count. While controlling for sociodemographics, multinomial regression modelling was used to examine associations between ACE count and neurodivergence status. Bivariate analyses using chi-squared tests were also used to explore associations between both neurodivergence status and ACE count and outcome measures including experiencing poor general health, low mental wellbeing, ever being arrested, and ever being incarcerated. Binary logistic regression models (enter method) were used to examine the independent relationships between both neurodivergence status and ACE count and outcomes including poor general health, low mental wellbeing, ever being arrested, and ever being incarcerated, while controlling for sociodemographics.

Multiplicative interaction terms are commonly utilised to assess the interaction of two risk factors on outcome variables; however, the absence of a significant effect does not mean that there is no relevant interaction [35, 36]. Additive interactions are also utilised to explore whether the combined effects of two risk factors together increases the risk of an outcome to a greater extent than adding the risk of the outcome from each risk factor individually. As such, the excess risk produced by combining the two risk factors can be calculated [35, 37]. The approach utilised in the current study is consistent with the analytic approach by Amos et al. [38], which adapted the methods of Andersson et al. [37], to calculate excess risk using estimated marginal means. While adjusting for age, sex, ethnicity, and deprivation, generalised linear models using binary logistic regression with an estimated marginal means function were utilised to identify the risks of poor general health, low mental wellbeing, ever being arrested, and ever being incarcerated for different combinations of neurodivergence and ACE count status (NT less than four ACEs (reference group), NT 4 + ACEs, ND less than four ACEs, ND 4 + ACEs). Estimated marginal means were first converted into percentages (risk). The risk for each outcome in the reference group was subtracted from the risk in all the other groups. The risk for each outcome in the two single risk factor groups (NT 4 + ACEs; ND less than four ACEs) were summed to give the expected risk of each outcome in the combined ND 4 + ACEs group. Expected risks for each outcome in the combined group was then subtracted from the observed risks in the combined group, leaving the excess risk of each outcome in the combined group, above what would be expected when summing the risks from the two single risk factor groups [38].

### Ethical approval

Ethical approval was granted for the study by Liverpool John Moores Research Ethics Committee (23/PHI/050).

## Results

### ND and sociodemographics

Overall, 10.3% (*n* = 528) of the sample indicated that they are ND, 5.8% (*n* = 295) were diagnosed, and 4.6% (*n* = 233) either self-diagnosed or suspected that they are ND. Table 1 shows the sociodemographic characteristics of ND and NT individuals. There were significant associations between neurodivergence status and sex (ND: female, 11.7%; male, 8.6%; *p* < 0.001), age group (ND: 18–24 years, 15.4%; 25–54 years, 14.7%; 55 + , 4.7%; *p* < 0.001), ethnicity (ND: any White background, 10.6%; any non-White background, 7.0%; *p* < 0.05), and deprivation quintile (ND: 1 most deprived, 12.3%; 2, 10.6%; 3, 9.4%; 4, 8.0%; 5 least deprived, 4.1%; *p* < 0.001).

**Table 1:** Sociodemographics by neurodivergence status

	**All % (*n*)**	**Neurodivergent % (*n*)**	**Neurotypical % (*n*)**	**χ**^**2**^	***p***
**Overall**	–	10.3 (528)	89.7 (4590)	–	–
**Sex**					
Male	47.4 (2553)	8.6 (207)	91.4 (2213)		
Female	52.6 (2828)	11.7 (314)	88.3 (2374)	13.263	< 0.001
**Age group (years)**					
18–24	9.5 (508)	15.4 (72)	84.6 (395)		
25–54	46.4 (2493)	14.7 (346)	85.3 (2000)		
55 +	44.1 (2369)	4.7 (107)	95.3 (2177)	141.432	< 0.001
**Ethnicity**					
Any White ethnic background	93.0 (4985)	10.6 (501)	89.4 (4246)		
Any non-White ethnic background	7.0 (377)	7.0 (25)	93.0 (333)	4.214	< 0.05
**Deprivation quintiles**					
1 (most deprived)	46.0 (2480)	12.3 (287)	87.7 (2041)		
2	15.8 (854)	10.6 (86)	89.4 (726)		
3	15.6 (840)	9.4 (76)	90.6 (733)		
4	15.5 (835)	8.0 (64)	92.0 (736)		
5 (least deprived)	7.2 (386)	4.1 (15)	95.9 (354)	31.218	< 0.001

### Neurodivergence and ACEs

In bivariate analyses, using chi-squared tests, there was a significantly higher prevalence of each ACE amongst ND individuals compared to NT individuals (Table 2). Amongst ND individuals, the most common ACE was verbal abuse (44.7%), while the least common ACE was household member incarceration (6.3%). Amongst NT individuals, the most common ACE was physical abuse (21.7%), while the least common ACE was household member incarceration (2.2%). 73.5% of ND individuals had experienced at least one ACE compared to 48.1% of NT individuals (Table 2).

**Table 2:** Prevalence of ACE types by neurodivergence status

	**All % (*n*)**	**Neurodivergent % (*n*)**	**Neurotypical % (*n*)**	**χ**^**2**^	***p***
Physical abuse	23.0 (1241)	38.3 (202)	21.7 (998)	71.034	< 0.001
Verbal abuse	23.4 (1265)	44.7 (236)	21.4 (981)	140.851	< 0.001
Sexual abuse	6.5 (352)	16.3 (86)	5.4 (249)	89.582	< 0.001
Household mental illness	15.5 (837)	38.3 (202)	13.1 (599)	226.019	< 0.001
Household alcohol harm	13.0 (704)	28.0 (148)	11.5 (529)	110.955	< 0.001
Household drug harm	4.0 (215)	11.4 (60)	3.1 (144)	81.598	< 0.001
Witnessing household violence	15.6 (841)	26.3 (139)	14.5 (667)	48.759	< 0.001
Household incarceration	2.7 (143)	6.3 (33)	2.2 (101)	28.889	< 0.001
Parental separation	20.2 (1090)	35.0 (185)	18.8 (864)	75.406	< 0.001
ACE count					
0 ACEs	50.2 (2708)	26.5 (140)	51.9 (2381)		
1 ACE	19.0 (1027)	18.0 (95)	19.7 (903)		
2–3 ACEs	18.9 (1021)	25.4 (134)	18.6 (856)		
4 + ACEs	11.8 (639)	30.1 (159)	9.8 (159)	237.740	< 0.001

In multivariate analyses, using multinomial regression, while controlling for sex, age, ethnicity, and deprivation quintile, compared to NT peers, ND individuals were nearly five times (AOR 4.89 (3.78–6.32); *p* < 0.001) more likely to experience 4 + ACEs than no ACEs, over twice (AOR 2.53 (1.96–3.27); *p* < 0.001) as likely to experience 2–3 ACEs than no ACEs, and over 1.6 times (AOR 1.66 (1.26–2.19); *p* < 0.001) more likely to experience 1 ACE than no ACEs.

### Neurodivergence, ACEs, and health and criminal justice outcomes

In bivariate analyses, using chi-squared tests, there were significant associations between neurodivergence status and experiencing poor general health (*p* < 0.001), low mental wellbeing (*p* < 0.001), ever being arrested (*p* < 0.001), and ever being incarcerated (*p* < 0.001), similarly there were significant associations between ACE count and experiencing poor general health (*p* < 0.01), low mental wellbeing (*p* < 0.001), ever being arrested (*p* < 0.001), and ever being incarcerated (*p* < 0.001; Table 3).

**Table 3:** Prevalence of being arrested, being incarcerated, experiencing poor general health, and experiencing low mental wellbeing, by neurodivergence status and ACE count

	**Poor general health % (*n*)**	**Low mental wellbeing % (*n*)**	**Ever been arrested % (*n*)**	**Ever been incarcerated % (*n*)**
All	19.0 (962)	14.1 (717)	8.6 (453)	5.2 (274)
Neurodivergence status				
Neurodivergent	29.2 (147)	28.6 (144)	17.3 (90)	8.8 (46)
Neurotypical	17.6 (767)	11.7 (513)	7.6 (341)	4.7 (213)
χ^2^	39.255	110.026	55.421	15.465
* p*	< 0.001	< 0.001	< 0.001	< 0.001
ACE count				
None	18.0 (444)	9.9 (246)	4.8 (125)	2.4 (64)
1 ACE	16.8 (166)	12.2 (122)	7.6 (77)	4.6 (47)
2–3 ACEs	21.4 (212)	19.8 (197)	13.6 (137)	8.6 (87)
4 + ACEs	23.1 (140)	24.2 (152)	18.1 (114)	12.0 (76)
χ^2^	14.861	118.717	154.124	125.392
* p*	0.002	< 0.001	< 0.001	< 0.001

In multivariate analyses, while controlling for sociodemographics, compared to NT peers, ND individuals were 2.42 times ((1.92–3.05); *p* < 0.001) more likely to experience poor general health. However, while ACE count significantly contributed to the model when predicting poor general health, those with higher ACE counts were not more likely to experience poor general health independent of neurodivergence status (Additional file 1: Table S1). In a separate model while controlling for sociodemographics, compared to NT peers, ND individuals were 2.34 times ((1.85–2.95); *p* < 0.001) more likely to experience low mental wellbeing. Compared to those with no ACEs, those with 2–3 ACEs (AOR 2.15 (1.73–2.68); *p* < 0.001) and 4 + ACEs (AOR 2.33 (1.82–2.99); *p* < 0.001) were significantly more likely to experience low mental wellbeing (Additional file 1: Table S2). In a separate model while controlling for sociodemographics, compared to NT peers, ND individuals were 2.37 times ((1.78–3.16); *p* < 0.001) more likely to ever be arrested. Compared to those with no ACEs, those with 1 ACE (AOR 1.56 (1.15–2.12); *p* < 0.01), 2–3 ACEs (AOR 2.81 (2.14–3.70); *p* < 0.001), and 4 + ACEs (AOR 4.43 (3.27–6.01); *p* < 0.001) were significantly more likely to ever be arrested (Additional file 1: Table S3). In a separate model while controlling for sociodemographics, compared to NT peers, ND individuals were 1.58 times ((1.09–2.29); *p* < 0.05) more likely to ever be incarcerated. Compared to those with no ACEs, those with 1 ACE (AOR 1.77 (1.18–2.63); *p* < 0.01), 2–3 ACEs (AOR 3.44 (2.42–4.88); *p* < 0.001), and 4 + ACEs (AOR 5.49 (3.75–8.04); *p* < 0.001) were significantly more likely to ever be incarcerated (Additional file 1: Table S4).

**Fig. 1:**
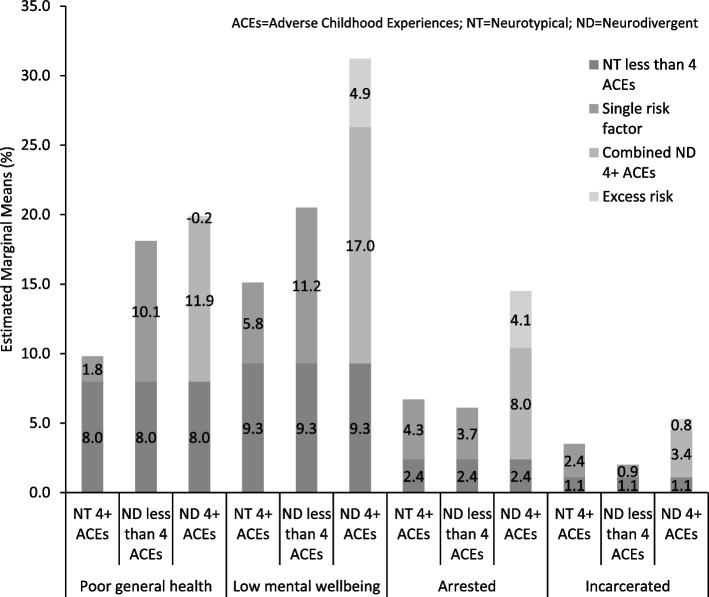
Additive effects of neurodivergence and ACE count as individual and combined risk factors for experiencing poor general health, low mental wellbeing, ever being arrested, and ever being incarcerated. Estimated marginal means are used to indicate the adjusted mean prevalence of each outcome measure by neurodivergence and ACE count status (i.e. NT and less than four ACEs, NT and 4 + ACES, ND and less than four ACEs, and ND and 4 + ACEs). The prevalence of each outcome in the NT less than four ACEs group is indicated by the bottom bar for each outcome (e.g. poor general health, 8.0%). The overall prevalence of an outcome in the NT 4 + ACEs, ND less than four ACEs, and the ND 4 + ACEs groups is taken by summing each bar for that group. The ‘single risk factor’ bar for the NT 4 + ACEs and ND less than four ACEs groups denotes the additional prevalence of an outcome experienced by groups with only one of the risk factors (only 4 + ACEs, or only ND), above the prevalence experienced by the group with neither risk factor (i.e. NT with less than four ACES). For the ND 4 + ACEs group, the ‘combined ND 4 + ACEs group’ prevalence denotes the expected prevalence; the ‘excess risk’ denotes the difference between the observed and expected prevalence of an outcome for this group. ACEs, adverse childhood experiences; NT, neurotypical; ND, neurodivergent

Overall, 8.0% of individuals in the NT with less than four ACEs group experienced poor general health. This increased above this baseline by 1.8% for those in the NT with four or more ACEs group and 10.1% in the ND with less than four ACEs group. Overall, 9.8% of individuals in the NT with less than four ACEs group experienced poor general health, compared to 18.1% in the ND with less than four ACEs group and 19.7% in the ND with four or more ACEs group. There was a very small decrease (− 0.2%) in the proportion of individuals experiencing poor general health in the ND with four or more ACEs group that was due to excess risk (Fig. 1).

Overall, 9.3% of individuals in the NT with less than four ACEs group experienced low mental wellbeing. This increased above this baseline by 5.8% for those in the NT with four or more ACEs group and 11.2% in the ND with less than four ACEs group. Overall, 15.1% of individuals in the NT with less than four ACEs group experienced low mental wellbeing, compared to 20.5% in the ND with less than four ACEs group and 31.2% in the ND with four or more ACEs group. There was a 4.9% rise in the proportion of individuals experiencing low mental wellbeing in the ND with four or more ACEs group that was due to excess risk (Fig. 1).

Overall, 2.4% of individuals in the NT with less than four ACEs group had ever been arrested. This increased above this baseline by 4.3% for those in the NT with four or more ACEs group and 3.7% in the ND with less than four ACEs group. Overall, 6.7% of individuals in the NT with less than four ACEs group had ever been arrested, compared to 6.1% in the ND with less than four ACEs group and 14.5% in the ND with four or more ACEs group. There was a 4.1% rise in the proportion of individuals who had ever been arrested in the ND with four or more ACEs group that was due to excess risk (Fig. 1).

Overall, 1.1% of individuals in the NT with less than four ACEs group had ever been incarcerated. This increased above this baseline by 2.4% for those in the NT with four or more ACEs group and 0.9% in the ND with less than four ACEs group. Overall, 3.5% of individuals in the NT with less than four ACEs group had ever been arrested, compared to 2.0% in the ND with less than four ACEs group and 5.3% in the ND with four or more ACEs group. There was a 0.8% rise in the proportion of individuals who had ever been arrested in the ND with four or more ACEs group that was due to excess risk (Fig. 1).

## Discussion

The aim of the current study was to explore the associations between neurodivergence status and adverse childhood experiences, and to explore their relative associations with health and wellbeing, and criminal justice outcomes using a representative general population sample. The current study has shown that in a general population sample from one English region, the ND population experiences a disproportionately high prevalence of ACEs. Critically, the prevalence of each individual ACE included in the current study was significantly higher amongst ND individuals compared to NT peers. Furthermore, ACE count was also significantly associated with ND status, with three in ten ND individuals experiencing four or more ACEs, compared to one in ten NT individuals; this relationship remained significant after controlling for sociodemographics. These findings are in line with findings from research with specific groups of neurodivergent individuals (i.e. autistic individuals and ADHD individuals) and individual types of ACEs, and the current study shows that ACEs are more prevalent amongst the ND population while including individuals self-diagnosing or suspecting of their neurodivergence [12–15]. The higher prevalence of ACEs amongst the ND population will have significant implications for the health and morbidity, social, educational, and offending outcomes of this group, with strong evidence which consistently links cumulative experiences of ACEs to an increased likelihood of experiencing a range of poorer outcomes across the lifecourse, with further implications for how individuals are able to access key social determinants of health [16–19]. The heightened levels of ACEs in this group may therefore help to explain, at least in part, the health inequalities that are experienced by the ND population, with higher levels of physical and mental health issues in this group [2–8].

Findings from the current study demonstrated that after controlling for sociodemographics, neurodivergence status and ACE count were independently associated with risks of experiencing low mental wellbeing, ever being arrested, and ever being incarcerated, while ND status but not ACE count was independently associated with poor general health, indicating that ND populations, regardless of their experiences of ACEs, are more likely to experience negative health, wellbeing, and criminal justice outcomes. Further, after adjusting for sociodemographics, the prevalence of experiencing poor general health, low mental wellbeing, ever being arrested, and ever being incarcerated was highest in the ND group with four or more ACEs and lowest in the NT group with less than four ACEs. Specifically, the prevalence of experiencing poor general health and low mental wellbeing were even higher in the ND group without four or more ACEs than the NT groups with or without four or more ACEs. When exploring the additive effects of combining being ND and experiencing four or more ACEs, there were excess risks of experiencing low mental wellbeing (4.9%), ever being arrested (4.1%), and a small excess risk of ever being incarcerated (0.8%), with a higher prevalence of each than would be expected by adding together the risks of each for those who are ND and those who experienced 4 + ACEs separately. However, there was no excess risk of experiencing poor general health when combining being ND and experiencing four or more ACEs with a smaller increase in risk than would be expected (− 0.2%). Therefore, findings in the current study may indicate that the combination of being ND and having experienced heightened levels of ACEs could additively increase risks of experiencing certain poor wellbeing and criminal justice outcomes (particularly low mental wellbeing and ever being arrested), to a greater extent than either being ND or experiencing high levels of ACEs alone, however, may not increase risks of other outcomes for example poor general health [23].

The higher than expected increase in prevalence of negative outcomes, in particular low mental wellbeing and ever being arrested, for those who are ND and have experienced heightened levels of ACEs could be explained by stress physiology [23]. ND individuals may experience a greater degree of stressors, particularly everyday experiences which are not experienced as stressful by NT individuals. This may contribute to a greater allostatic load for neurodivergent individuals, compared to their NT peers, increasing the likelihood of negative wellbeing and criminal justice outcomes to a greater extent than would be expected when ND individuals experience a high number of ACEs [22, 23]. This potential increased exposure to stressors for ND individuals could also help to explain some of the relationships seen in the current study between neurodivergence status and for example, experiencing poor general health. While previous research has shown that individuals who experience more ACEs are more likely to experience poor general health, these studies did not account for neurodivergence status [16, 18]. In the current study, ND individuals were more likely to experience poor general health than NT individuals, independent of ACE count. Additionally, having a greater ACE count did not significantly increase the risks of experiencing poor general health, independently of neurodivergence status. As such, associations found in previous research between experiencing poor general health and experiencing ACEs may be led primarily by the ND population. This could also explain why when combining being ND and experiencing four or more ACEs there is a smaller increase in the risks of experiencing poor general health that would be expected, as being ND may have such a substantial impact on the risks of experiencing poor general health that experiencing ACEs has a diminished additional impact on the risks of experiencing poor general health. However, this would need to be explored further in future research. Findings in the current study could be explained by stress physiology, but there are likely to be other contributing factors also, such as reduced access for ND populations compared to NT populations to key social determinants of health (e.g. fulfilling employment and education), reduced access to healthcare services or other community-level supports, reduced levels of positive resilience factors in childhood or adulthood, or increased levels of engagement in health risk behaviours [4, 6, 8, 39–41]. Future research should aim to explore the pathways that lead to heightened levels of poor health, wellbeing, and criminal justice outcomes in ND populations.

Findings in the current study suggest that ND individuals may have an increased level of engagement with a range of different health, mental health, police, and criminal justice services. As such, these services must be equipped with the knowledge and skills to adequately meet the needs of ND individuals, both in interacting with their services in the first instance, and to address their presenting issues. However, evidence from both the United Kingdom (UK) and internationally indicates that some services are not currently meeting the needs of ND populations both in childhood and adulthood, due to inaccessibility (including services not catering to ND individuals’ sensory needs), long wait times, lack of screening for different types of neurodivergence, diagnostic overshadowing, only offering support on the basis of diagnosis—rather than needs, and that engagement with or waiting for services can lead to further traumatisation [39, 41–45]. Further, evidence from health, mental health, police, and criminal justice services indicates that practitioners may not currently have the tools, knowledge, or skills to meet the needs of different ND groups [41, 43–47].

Across the UK and internationally, different services are implementing training sessions and programmes of work in order to work more effectively with individuals who have faced heightened levels of trauma [16]. Evidence from training programmes implemented with services in the UK indicates that such programmes can be effective in improving staff’s trauma-informed knowledge and attitudes, and confidence to interact effectively with people who have experienced ACEs [48–50]. However, due to the higher than expected prevalence of certain negative outcomes for those who are ND and have experienced heightened levels of ACEs, programmes of work at service and systems-levels to improve trauma-informed working that do so without consideration of the prevalence and impacts of trauma amongst ND populations may not be effective.

The current research put into wider context indicates that there is a need to improve the knowledge and skills of health, mental health, police, and criminal justice practitioners on the needs of ND populations with and without heightened levels of ACEs. This is critical as each contact with services acts as an opportunity to better understand ND individuals and offer support which may reduce their heightened experiences of poorer health, wellbeing, and criminal justice outcomes compared to NT peers. Importantly however, there is a need for multiple agencies that work with ND children and adults in the general population to implement and evidence the impact of programmes and policies which aim to prevent and mitigate the impacts of ACEs specifically amongst ND populations, whether diagnosed or not. This may help to prevent longer-term negative health, wellbeing, and criminal justice outcomes for ND populations in the first instance, rather than waiting to respond to negative outcomes, including crisis points, in this group when they occur.

### Limitations

Findings in the current study must be considered in light of the following limitations.

The current study does not differentiate between different groups of ND individuals, as such findings in the current study may apply differently to different ND groups. Future research should aim to collect data on the types of ND diagnoses which individuals have, to explore whether ACEs are more likely amongst certain ND groups than others, and whether ACEs have differential impacts on health, wellbeing, and criminal justice outcomes dependent on types of neurodivergence.

Utilising household survey methodology while useful for providing a representative general population sample is not without limitations. For example, it is possible that this methodology may not be able to capture the experiences of all ND individuals, particularly for ND people with more significant differences in verbal and written communication. This study was also unable to explore if there were differences between non-respondents and respondents on key sociodemographic factors. As such, findings may not be applicable to all ND populations. However, this limitation may have been somewhat mitigated by the option for participants to complete online in their own time. Future research should aim to explore what are the best methodologies for understanding the prevalence of ACEs amongst ND people in the general population who may communicate differently.

The current study utilised retrospective self-reporting of ACEs and other outcome measures, meaning that there may be some element of recall bias in reporting of events which happened in childhood, particularly for older individuals. This may under-estimate the true prevalence of ACEs. Further, it should be noted that as ‘prefer not to say’ in the current study was coded as ‘no’ for all ACEs measures, the prevalence of ACEs are considered a minimum. However, utilising retrospective self-reporting remains a valid way of measuring ACEs in general population samples and does not suffer from the same limitations as studies which utilise reporting by others such as parents or professionals, or studies conducted when the individual of interest is still a child. These studies may also under-estimate the true prevalence of ACEs, particularly for ACEs such as abuse and neglect which may be underreported.

The use of ACE count as a measure is also subject to limitations, for example, having an exposure to an ACE does not account for the frequency, severity, or duration of the ACE exposure. The frequency, severity, or duration of exposure to an ACE may have implications for the relationships between ACE exposures and negative outcomes. While the evidence on cumulative exposures to ACEs and harmful outcomes is strong, currently there is less evidence about the extent to which more severe or repeated ACE exposures may have more detrimental impacts than a single incident of exposure to an ACE. Future research should aim to explore relationships between neurodivergence status and the frequency of exposure to different types of ACEs, and how this may impact on the risks of experiencing negative health, wellbeing, and criminal justice outcomes across the lifecourse.

Given the cross-sectional and retrospective nature of the study data, the findings of the current study should not be interpreted as causal in any direction in terms of the relationships shown between neurodivergence status and heightened experiences of ACEs, and negative outcomes. Future research utilising prospective methods with children should be a priority; however, care needs to be taken given that neurodivergent diagnoses generally do not happen until children reach certain age milestones, and often not until later, despite always experiencing life as a ND individual. Further, care needs to be taken in utilising prospective methodologies when examining the longer-term impacts of ACEs, given that there is a duty to protect a child if ACEs are identified, and that any intervention may in turn diminish the impacts of experiencing ACEs.

## Conclusions

In a general population sample from an English region, a significantly higher proportion of ND individuals experienced each ACE, and had a greater number of ACEs overall, compared to their NT peers. Independent of ACE count, ND individuals were more likely to experience poor general health, low mental wellbeing, be arrested, and be incarcerated. Even when compared to NT peers with four or more ACEs, there was a higher prevalence of experiencing poor general health and low mental wellbeing amongst ND individuals without four or more ACEs, and a higher prevalence of poor general health, low mental wellbeing, ever being arrested, and ever being incarcerated amongst ND individuals with four or more ACEs. Findings in the current study may indicate that the combination of being ND and having experienced heightened levels of ACEs could additively increase risks of experiencing certain poor wellbeing and criminal justice outcomes (particularly low mental wellbeing and ever being arrested), with a higher prevalence of these outcomes than would be expected amongst this group, however, may not increase risks of other outcomes for example poor general health. Interestingly, findings in the current study were not limited to ND people who had received a diagnosis, but also included self-diagnosed individuals or those suspecting that they are ND. Preventing and responding to ACEs amongst ND populations should become a priority in order to reduce the risks of experiencing poor health, wellbeing, and criminal justice outcomes in this population. Further, there is a need to upskill health and wellbeing, and police and criminal justice workforces in how to most effectively work with ND individuals, and particularly ND individuals with high levels of ACEs.

## Supplementary Information

Additional file 1: Table S1 Multivariate relationships between neurodivergence and ACE count and poor general health. Table S2 Multivariate relationships between neurodivergence and ACE count and low mental wellbeing. Table S3 Multivariate relationships between neurodivergence and ACE count and ever being arrested. Table S4 Multivariate relationships between neurodivergence and ACE count and ever being incarcerated.

## Data Availability

The study materials and data will not be made available publicly or on request, due to a need to protect the privacy of participants.

## Abbreviations

ND: Neurodivergent
NT: Neurotypical
ADHD: Attention deficit hyperactivity disorder
OCD: Obsessive compulsive disorder
ACEs: Adverse childhood experiences
LSOAs: Lower Super Output Areas
IMD: English Index of Multiple Deprivation
BRFSS: Behavioral Risk Factor Surveillance System
SWEMWBS: Short Warwick-Edinburgh Mental Wellbeing Scale
UK: United Kingdom
