# Supplementary material for: Relationships between neurodivergence status and adverse childhood experiences, and impacts on health, wellbeing, and criminal justice outcomes: findings from a regional household survey study in England
Source: BMC Med. 2024 Dec 18;22:592. doi: 10.1186/s12916-024-03821-1 (PMC11657135; doi:10.1186/s12916-024-03821-1)
Supplement: Supplementary file 1 — Additional file 1: Table S1 Multivariate relationships between neurodivergence and ACE count and poor general health. Table S2 Multivariate relationships between neurodivergence and ACE count and low mental wellbeing. Table S3 Multivariate relationships between neurodivergence and ACE count and ever being arrested. Table S4 Multivariate relationships between neurodivergence and ACE count and ever being incarcerated. [file 12916_2024_3821_MOESM1_ESM.docx]

**Table S1: Multivariate relationship between experiencing poor general health and neurodivergence and ACE count**

| Model 1 | | |
| --- | --- | --- |
|  | Adjusted odds ratio (AOR) | Significance *(p)* |
| **Sex** |  |  |
| Female (ref) |  |  |
| Male | 0.85 (0.73-0.99) | 0.033 |
| **Age (years)** |  |  |
| 18-24 (ref) |  | <0.001 |
| 25-54 | 2.01 (1.38-2.93) | <0.001 |
| 55+ | 5.78 (3.97-8.42) | <0.001 |
| **Ethnicity** |  |  |
| Any other ethnic background (ref) |  |  |
| White | 1.34 (0.94-1.92) | 0.110 |
| **Deprivation quintile** |  |  |
| 5 (least deprived) (ref) |  | <0.001 |
| 1 (most deprived) | 3.81 (2.62-5.55) | <0.001 |
| 2 | 2.07 (1.37-3.11) | <0.001 |
| 3 | 1.60 (1.05-2.42) | 0.029 |
| 4 | 1.59 (1.05-2.41) | 0.028 |
| **Neurodivergence status** |  |  |
| Neurotypical (ref) |  |  |
| Neurodivergent | 2.42 (1.92-3.05) | <0.001 |
| **ACE count** |  |  |
| None (ref) |  | 0.035 |
| 1 ACE | 0.88 (0.72-1.09) | 0.242 |
| 2-3 ACEs | 1.18 (0.97-1.44) | 0.099 |
| 4+ ACEs | 1.24 (0.97-1.57) | 0.085 |

**Table S2: Multivariate relationship between experiencing low mental wellbeing and neurodivergence and ACE count**

| Model 1 | | |
| --- | --- | --- |
|  | Adjusted odds ratio (AOR) | Significance *(p)* |
| **Sex** |  |  |
| Female (ref) |  |  |
| Male | 0.92 (0.77-1.09) | 0.328 |
| **Age (years)** |  |  |
| 18-24 (ref) |  | 0.158 |
| 25-54 | 0.80 (0.60-1.06) | 0.122 |
| 55+ | 0.92 (0.69-1.24) | 0.600 |
| **Ethnicity** |  |  |
| Any other ethnic background (ref) |  |  |
| White | 0.84 (0.61-1.16) | 0.292 |
| **Deprivation quintile** |  |  |
| 5 (least deprived) (ref) |  | <0.001 |
| 1 (most deprived) | 2.74 (1.76-4.27) | <0.001 |
| 2 | 1.70 (1.05-2.75) | 0.031 |
| 3 | 1.45 (0.89-2.37) | 0.136 |
| 4 | 1.22 (0.74-2.01) | 0.436 |
| **Neurodivergence status** |  |  |
| Neurotypical (ref) |  |  |
| Neurodivergent | 2.34 (1.85-2.95) | <0.001 |
| **ACE count** |  |  |
| None (ref) |  | <0.001 |
| 1 ACE | 1.28 (1.00-1.63) | 0.050 |
| 2-3 ACEs | 2.15 (1.73-2.68) | <0.001 |
| 4+ ACEs | 2.33 (1.82-2.99) | <0.001 |

**Table S3: Multivariate relationship between being arrested and neurodivergence and ACE count**

| Model 1 | | |
| --- | --- | --- |
|  | Adjusted odds ratio (AOR) | Significance *(p)* |
| **Sex** |  |  |
| Female (ref) |  |  |
| Male | 6.02 (4.68-7.75) | <0.001 |
| **Age (years)** |  |  |
| 18-24 (ref) |  | <0.001 |
| 25-54 | 2.91 (1.75-4.86) | <0.001 |
| 55+ | 2.99 (1.78-5.04) | <0.001 |
| **Ethnicity** |  |  |
| Any other ethnic background (ref) |  |  |
| White | 1.78 (1.07-2.96) | 0.027 |
| **Deprivation quintile** |  |  |
| 5 (least deprived) (ref) |  | <0.001 |
| 1 (most deprived) | 2.18 (1.31-3.65) | 0.003 |
| 2 | 1.42 (0.81-2.48) | 0.220 |
| 3 | 1.47 (0.84-2.57) | 0.182 |
| 4 | 0.95 (0.53-1.71) | 0.859 |
| **Neurodivergence status** |  |  |
| Neurotypical (ref) |  |  |
| Neurodivergent | 2.37 (1.78-3.16) | <0.001 |
| **ACE count** |  |  |
| None (ref) |  | <0.001 |
| 1 ACE | 1.56 (1.15-2.12) | 0.005 |
| 2-3 ACEs | 2.81 (2.14-3.70) | <0.001 |
| 4+ ACEs | 4.43 (3.27-6.01) | <0.001 |

**Table S4: Multivariate relationship between being incarcerated and neurodivergence and ACE count**

| Model 1 | | |
| --- | --- | --- |
|  | Adjusted odds ratio (AOR) | Significance *(p)* |
| **Sex** |  |  |
| Female (ref) |  |  |
| Male | 7.44 (5.29-10.48) | <0.001 |
| **Age (years)** |  |  |
| 18-24 (ref) |  | <0.001 |
| 25-54 | 4.13 (1.98-8.62) | <0.001 |
| 55+ | 3.40 (1.61-7.18) | 0.001 |
| **Ethnicity** |  |  |
| Any other ethnic background (ref) |  |  |
| White | 2.02 (1.04-3.93) | 0.039 |
| **Deprivation quintile** |  |  |
| 5 (least deprived) (ref) |  | <0.001 |
| 1 (most deprived) | 2.52 (1.25-5.08) | 0.010 |
| 2 | 1.97 (0.93-4.15) | 0.076 |
| 3 | 1.74 (0.82-3.71) | 0.152 |
| 4 | 0.91 (0.41-2.06) | 0.829 |
| **Neurodivergence status** |  |  |
| Neurotypical (ref) |  |  |
| Neurodivergent | 1.58 (1.09-2.29) | 0.016 |
| **ACE count** |  |  |
| None (ref) |  | <0.001 |
| 1 ACE | 1.77 (1.18-2.63) | 0.005 |
| 2-3 ACEs | 3.44 (2.42-4.88) | <0.001 |
| 4+ ACEs | 5.49 (3.75-8.04) | <0.001 |
